# Home-Related Injuries: Do Pay Much Attention to Traffic Accidents Resulted in Home-Related Injuries Negligence?

**DOI:** 10.5812/atr.10270

**Published:** 2013-02-01

**Authors:** Mohammad Reza Fazel

**Affiliations:** 1 Trauma Research Center, Kashan University of Medical Sciences, Kashan, IR Iran

**Keywords:** Home, Injury, Trauma

Trauma as the sixth leading cause of death, and the fifth cause of moderate and severe disability around the world, accounts for 10% of mortalities (1). Although almost all sorts of traumas are common, focused on traffic accidents, which may be due to the rapid growth of accidents or worldwide social, economic, political and health problems, have resulted in ignoring the others especially household events, which may involve every member of the family. In the united states, house-related injuries are the main causes of injury among young children, which is accounting for more than 13 million outpatient visits, 4 million emergency visits, 74000 hospitalizations, and 2800 deaths each year (2). In the United Kingdom, 75 children under 15 years old, died due to injuries in the home a year. This figure comprises 25 percent of all child injury deaths (3). In Iran, during recent decades, rapid changes have appeared in different aspects of social life and living style from a traditional pattern to a modern one. Living in an apartment has become common and numerous changes have made in the internal design and decoration of houses. These are sometimes different with the traditional cultural beliefs, trainings and behavioral habits, and may be resulted in changes in the pattern and incidence of the injuries. In spite of these changes, injuries in the home environment have remained largely neglected or conceivably no systematic studies have been conducted on these cases. Another persisting worry is the fact that it is mainly under-estimated in the shadow of traffic and work-environment injuries. In a study in Tehran (4) home environmental injuries is the second cause of trauma (38%) placed after traffic accident (45%) among children while females are at high risk of home-related injuries. The most common mechanism of injuries was falling. In a study conducted by Fazel et al. in Kashan (5) during a six-year period, 25.2% of all injuries happened at home. While most of the reported injuries in males were seen under the age of 15 years old, it was reported that in females the injuries were more common after the age of 55 years. Falling was the most common mechanism of injury followed by sharp-object injuries, in this study. In another study by Mohammadi et al. (6) burning with hot liquids (29%), injuries with sharp instruments (24%) and falling (11%) were the common causes of home-related traumas. Major causes leading to death were burns (incidence rate of 0.15 per 10000 population) and falls (0.05 per 10000 population). Children under 5 years old formed the largest group of home-related injured people. Two important points of the above findings are 1 - home-related injury is a major risk for family health, 2- the victims of the mentioned injuries are the innocent children, fragile elder people and housewives who have the least responsibility in the construction of the houses and management of their safety measurements. The arrangement of systematic approaches to study, arrange, and deliver strategies to reduce home accidents in our country is fundamental. These strategies should include the following items:

1- Modifying the home environment by the establishment of safety measures. 

2- Making changes in the structural design and construction techniques complying with cultural and traditional concepts of the society to increase safety at home. (supporting innovations in order to design safe homes)

3- Educational interventions with the aim of parental knowledge promotion by training them to use safety measures.

It is apparent that such strategies must also accompany compulsory rules in order to build a more secure environment by installing safety measures for the prevention of the injuries. To achieve these objectives, trauma research centers with a specific subgroup or committee considering home injuries can support the aforementioned ideas.
